# Effect of perceived distance to health facility on antenatal care service use in Sub-Saharan Africa: Do socio-demographic characteristics modify these associations?

**DOI:** 10.1177/17455057261446938

**Published:** 2026-05-13

**Authors:** Yibeltal Bekele, Ruth L Ngoma, Gedefaw Abeje, Bircan Erbas, Mehak Batra

**Affiliations:** 1 School of Psychology and Public Health, 2080 La Trobe University, Melbourne, VIC, Australia; 2 Department of Reproductive Health and Population Studies, School of Public Health, College of Medicine and Health Sciences, 128158 Bahir Dar University, Bahir Dar, Ethiopia; 3 Department of Rural Health Sciences, 2080 La Trobe University, Shepparton Campus, Shepparton, VIC, Australia; 4 School of Nursing and Midwifery, College of Health, Medicine and Wellbeing, Callaghan Campus, University of Newcastle, Newcastle, NSW, Australia

**Keywords:** perceived distance, antenatal care, ANC utilisation, sub-Saharan Africa

## Abstract

**Background:**

Although perceived distance is a key factor in deciding and reaching healthcare, its impact on antenatal care (ANC) utilisation remains underexplored.

**Objective:**

This study aims to examine the effect of perceived distance on ANC uptake, stratified by key maternal characteristics.

**Design:**

Cross-sectional analysis based on demographic and health survey data (DHS).

**Methods:**

This study analysed the demographic and health survey data of 26 sub-Saharan African countries, comprising 186,873 women who had given birth within the five years preceding the surveys. The exposure variable was perceived distance to a healthcare facility, categorised as “a big problem” or “not a big problem.” Whereas the outcome variable was the number of ANC contacts, classified as no, one to three, four to seven and eight or more contacts. A Generalised Structural Equation Model (GSEM) with a multinomial logit link was employed to examine the association. Analyses were further stratified by socio-demographic characteristics.

**Results:**

The analysis revealed that women who perceived the distance as a major problem had 15% higher odds of receiving only 1–3 contacts (aOR = 1.15, 95% CI: 1.05, 1.25, p = 0.002), and 51% higher odds of receiving no ANC (aOR = 1.51, 95% CI: 1.35, 1.66, p < 0.001), compared to those receiving eight or more contacts. These associations were particularly pronounced among women with lower educational status (aOR=1.48, 95%CI: 1.29, 1.64), rural residents (aOR=1.55, 95%CI: 1.37, 1.74), low household income (aOR=1.47, 95%CI: 1.27,1.68), and younger age (aOR=1.55, 95%CI: 1.31, 1.80).

**Conclusion:**

Perceived distance remains a significant barrier to the utilisation of antenatal care services in resource-limited settings. Strengthening health system responsiveness and addressing structural barriers, such as transportation infrastructures, through innovations like mobile antenatal care is vital to improving maternal health outcomes and advancing global health equity.

## Plain language summary

Ensuring access to high-quality antenatal care (ANC) is essential for improving maternal and newborn health outcomes. However, evidence on the effect of geographical distance on ANC use in sub-Saharan Africa remains limited and inconclusive. This study examined the relationship between perceived distance to a health facility and ANC utilisation across 26 sub-Saharan African countries. We analysed the Demographic and Health Survey (DHS) data from 186,873 women who had given birth in the five years preceding the survey. Women who reported that distance to a health facility was a major problem had 15% higher odds of attending only 1-3 ANC contacts and 51% higher odds of receiving no ANC, compared with women who attended at least eight contacts. These associations were stronger among women with lower levels of education, those living in rural areas, women from low-income households, and younger women. These findings underscore the importance of addressing perceived distance-related barriers to improve uptake of the WHO-recommended number of ANC contacts and to promote equitable access to maternal health services in sub-Saharan Africa.

## Introduction

ANC is a key maternal health platform delivered by healthcare professionals, including midwives, nurses, and doctors. It encompasses a wide range of interventions, such as maternal and fetal health assessments (e.g., screening for pre-eclampsia), nutritional support (e.g., iron and folic acid supplementation), and preventive measures like tetanus toxoid vaccination.^1^ Historically, the World Health Organisation (WHO) recommended at least four ANC contacts to reduce maternal and neonatal risks. However, in 2016, this recommendation was revised to a minimum of eight contacts to further reduce the risk of stillbirth and complications, while improving the overall experience of care.^2^ Evidence suggests that implementing this updated model can avert approximately eight perinatal deaths per 1,000 live births.^2^ Even attending just one ANC visit reduces the probability of adverse birth outcomes, such as low birth weight and neonatal mortality, with four or more contacts conferring additional survival benefits.^3^

More than 700 women died each day from preventable causes related to pregnancy and childbirth, with over 90% of these maternal deaths occurring in low- and middle-income countries in 2023. Sub-Saharan Africa alone accounted for approximately 70% of the global maternal mortality burden.^4^ This persistently high maternal mortality reflects stark global inequalities in access to quality health services and underscores broader socio-economic disparities across countries.^5^ The majority of maternal deaths worldwide are attributed to haemorrhage (27%), indirect obstetric causes such as pre-existing medical conditions (23%), and hypertensive disorders (16%).^5^ Maternal mortality is disproportionately higher among women living in poverty, those with low levels of education, residents of rural areas, and individuals at the extremes of reproductive age, both younger and older mothers.^5,6^ High-quality antenatal care (ANC) plays a critical role in reducing maternal mortality and morbidity, prematurity and intra-uterine fetal death by providing integrated healthcare services, facilitating health education, and enabling the early detection and management of pregnancy complications.^6^

Despite its proven benefits, ANC uptake remains low and varies widely by region and demographic characteristics. Globally, approximately 70% of pregnant women attend at least four ANC contacts, with higher coverage in urban areas (79.4%) compared to rural settings (60.2%). Regional disparities are also pronounced, with coverage particularly low in South Asia (58.4%) and sub-Saharan Africa (57.7%).^7^ Socio-economic and educational inequalities further exacerbate these gaps^8–11^; in sub-Saharan Africa, only 37.7% of women from the poorest households and 37.9% of those without formal education complete at least four ANC contacts.^12^ Uptake of the WHO-recommended eight or more contacts is even lower, just 11.3% in South Asia and 8.9% in sub-Saharan Africa.^13^

These disparities are driven by a combination of systemic and contextual barriers, including limited healthcare infrastructure, shortages of skilled providers, and inadequate transportation.^14–16^ Among these factors, perceived distance to healthcare facilities is particularly important, acting as a proxy for multiple overlapping challenges. Distance reflects not only physical accessibility but also economic constraints (e.g., high transport costs), infrastructural limitations (e.g., poor road networks), and socio-cultural barriers such as restrictions on women’s mobility in patriarchal contexts.^12,14,17^ Consequently, perceived distance serves as a broader indicator of healthcare accessibility, particularly for vulnerable groups such as women with low income, limited education, or those living in remote rural areas.

In sub-Saharan Africa, access to ANC is often further hindered by entrenched patriarchal norms and inadequate transportation infrastructure.^18,19^ Studies consistently identify distance as a significant barrier: for example, 28% of women in Ghana report distance as a major challenge, while over one-third of women in sub-Saharan Africa and South Asia cite distance as a barrier to accessing maternal healthcare services.^12,17,20^ However, evidence on the relationship between perceived distance and ANC utilisation remains inconclusive. While some studies report a strong negative association between distance and attending at least four contacts,^21,22^ others find no significant link when considering the updated WHO benchmark of eight or more contacts.^23,24^ Such inconsistencies may stem from methodological variations, including the binary categorisation of ANC use, low event proportions, or failure to consider intermediate utilisation patterns. Moreover, limited research has explored how the influence of distance varies across disadvantaged subgroups, such as women with low education, poor household wealth, young maternal age, or rural residence, who consistently exhibit the lowest levels of ANC coverage. Addressing these gaps requires both a more nuanced classification of ANC utilisation and an equity-focused lens.

By categorising ANC contacts into four groups: none, 1–3, 4–7, and ≥8 contacts, our analysis captures both historical and current WHO recommendations while providing greater insight into care-seeking patterns. Additionally, stratified multinomial analyses across key socio-demographic subgroups identify which populations are most affected by accessibility barriers. The findings provide actionable evidence to guide targeted interventions, such as community health worker outreach, transportation support, and mobile ANC services, aimed at improving equitable access to maternal healthcare in resource-limited settings.

## Methods

This study is a cross-sectional secondary data analysis of Demographic Health Survey (DHS) data conducted between 2015 and 2023 from 26 sub-Saharan African countries. DHS surveys are nationally representative, community-based surveys conducted roughly every five years in more than 90 low and middle-income countries. The DHS program collects harmonised data on maternal health, child health, nutrition, HIV/AIDS, and malaria. Further details about the program can be found on the DHS webpage (https://dhsprogram.com/). The original survey received ethical approval from both the ICF Institutional Review Board (IRB) and the relevant national IRBs. Further approval was obtained from La Trobe University with an ethical application number HEC23324. Moreover, formal approval was obtained from the DHS program. Informed verbal consent was obtained from all participants, including mothers aged between 15 and 17 years, who were considered emancipated minors. This study was reported in accordance with the appropriate STROBE guidelines.^25^

The DHS employed a two-stage stratified random sampling technique. In the first stage, enumeration areas (EA) were selected with probability proportional to size. In the second stage, households within the selected EAs were systematically sampled. A total of 186,873 women aged 15 to 49 years who had given birth within the five years preceding the survey were included in this analysis.

### Data collection

Data were collected through maternal recall using standardised questionnaires translated into local languages. Trained data collectors and supervisors, fluent in the local language, conducted the interviews. Data quality was ensured through a multilevel supervision system, including routine field monitoring and periodic contacts by central office staff.^26^

### Outcome variable

The primary outcome was antenatal care (ANC) service utilisation, categorised as none, one to three contacts, four to seven contacts, or eight or more contacts. This information was obtained through two questions: (1) “Did you see anyone for antenatal care for this pregnancy?” and (2) “How many times did you receive antenatal care during this pregnancy?” According to the WHO, pregnant women are recommended to receive a minimum of eight ANC contacts during pregnancy.^2^

### Exposure variable

The exposure variable of the study was perceived distance to the nearest health care facilities, classified as “a big problem” or “not a big problem”. This was assessed through the question: “When you want to get medical advice or treatment, is the distance to the health facility a big problem or not a big problem?” Both exposure and outcome variables were collected via maternal recall.

### Other variables

Variable selection was guided by Aderson’s Behavioural Model of Health Services Use and the Three Delay Model to ensure theoretical and causal relevance.^27,28^ According to Aderson’s model, determinants of health services utilisation are grouped into predisposing, enabling, and need factors. Predisposing factors included maternal age, maternal and partner education, parity, marital status, and maternal occupation. Enabling factors comprised household income, place of residence, and media exposure. Need-related factors such as medical health problems and pregnancy complications. A directed acyclic graph (DAG)^29^ was constructed to identify potential confounders and appropriate adjustment variables in the association between perceived distance to the health facility (exposure) and ANC utilisation (outcome). Based on these, the following variables were considered as a potential covariate: residence, maternal age, maternal occupation, maternal education, partner education, parity, household income, media exposure and marital status (Figure S1). Variables such as pre-existing medical conditions and pregnancy-related complications were not available in the dataset, and this limitation has been acknowledged in the limitations section.

### Statistical analysis

Descriptive statistics were used to summarise the exposure, outcome, and covariates. Group differences were assessed using the chi-square test, as all variables were categorical. To account for the hierarchical structure of the DHS data and the categorical nature of the outcome variable, a Generalised Structural Equation Model (GSEM) with a multinomial logit link was employed. Compared with conventional regression approaches, GSEM allows simultaneous estimation of multiple relationships while appropriately modelling correlated observations within complex survey data structures. The outcome variable, ANC utilisation, was classified into four categories: no contacts, 1–3 contacts, 4–7 contacts, and ≥8 contacts (reference category).^2^ Sample weights were applied to adjust for non-response and the DHS sampling design. A clustering variable (enumeration area) was included as a random effect to account for intra-cluster correlation. The model was adjusted for key covariates, including residence, maternal age, maternal occupation, maternal education, partner’s education, parity, household income, media exposure, and marital status.

Stratified analysis was conducted to explore subgroup-specific effects regardless of a significant interaction term, allowing for a more nuanced understanding of how key socio-demographic factors may influence the pathways to association, as previous theory shows that socio-demographic factors influenced the uptake of ANC services. Stratified analyses were conducted by maternal age, residence, maternal education, partner education, and household wealth to explore effect modification. These stratified models were not intended for additional confounding adjustment but to explore whether the association between perceived distance and ANC utilisation differed across subgroups. For each stratified variable, separate GSEMs were fitted within strata, and interaction terms between perceived distance and ANC utilisation across each subgroup. For each stratifying variable, separate GSEMs were fitted. Results were reported as adjusted odds ratios (aORs) with 95% confidence intervals (CIs). Statistical analyses were performed using STATA 18,^30^ with p-values ≤ 0.05 considered statistically significant. Multicollinearity was assessed using variance inflation factors (VIF), with all values below 5, indicating no evidence of multicollinearity. Model fit was evaluated using the Akaike information criterion (AIC), Bayesian Information Criteria (BIC), and log-likelihood values. The full model demonstrated a better fit, as evidenced by lower AIC, BIC and log-likelihood values compared to the null model.

## Results

Marked disparities in ANC utilisation were observed across socio-demographic subgroups (Table 1). Overall, among 186,873 women, 30.3% (n=55,932) received only 1–3 ANC contacts, 52.1% (n=96,239) received 4–7 contacts, and just 8.0% (n=14,823) achieved the recommended eight or more contacts.

**Table 1.** Descriptive characteristics of women by antenatal care (ANC) utilisation status in sub-Saharan Africa (n = 186,873).

Exposures		Antenatal care services utilisation				
		No (%)	1 to 3 contacts (%)	4 to 7 contacts (%)	At least eight contacts (%)	p-value
		(n=18,461)	(n=57,040)	(n=97,125)	(n=14,247)	
Perceived distance to the nearest healthcare n=179,162	Not a big problem	8041(7.35)	31,620(28.89)	60,038(54.85)	9763(9.92)	<0.001
	A big problem	10,097(14.49)	23,281(33.40)	32,426 (46.52)	3896(5.59)	
Age n=186,873	15-19	1501(10.51)	4901(34.32)	7150 (50.07)	727(5.09)	<0.001
	20-24	3792 (8.92)	13,345(31.40)	22,6267(53.24)	2738(6.44)	
	25-29	4477 (9.62)	13,653(29.33)	24,778(53.81)	3635(7.81)	
	30-34	3471(9.19)	11,100(29.38)	19,878 (52.62)	3327(8.81)	
	35-39	2844 (10.20)	8397(30.11)	14,151 (50.74)	2498 (8.96)	
	40-44	1655 (12.31)	4193(31.20)	6540(48.66)	1053 (7.83)	
	45-49	721 (16.23)	1451(32.66)	2002(45.06)	269(6.05)	
Educational status n=186,873	Illiterate	12,793 (19.08)	22,360(33.35)	28,833(43.01)	3059 (4.56)	<0.001
	Primary	3,818 (6.32)	21,683(35.89)	32,128 (53.18)	2785 (4.61)	
	Secondary	1,765(3.46)	11,891(23.33)	30,937 (60.70)	6373 (12.50)	
	Higher	85 (1.01)	1106 (13.09)	5227 (61.87)	2030 (24.03)	
Wealth index n=186,873	Poorest	8495 (18.34)	15,780(34.07)	20,217(43.64)	1831 (3.95)	<0.001
	Poorer	4854 (12.08)	13,223(32.90)	19,908 (49.54)	2202 (5.48)	
	Middle	2824 (7.56)	11,823(31.64)	20,033 (53.60)	2692 (7.20)	
	Richer	1,558 (4.66)	9606(28.70)	19,058(56.94)	3246(9.70)	
	Richest	730 (2.47)	6608(22.38)	17,909(60.66)	4276 (14.48)	
Residence n=186,873	Urban	3099 (5.05)	15,098(24.58)	35,366(57.58)	7855(12.79)	<0.001
	Rural	15,362(12.25)	41,942 (33.43)	61,759 (49.23)	6392 (5.10)	
Maternal working n=186,873	Yes	9701 (8.32)	35,239(30.24)	61,494(52.77)	10,108 (8.67)	<0.001
	No	8760 (12.46)	21,801(31.00)	35,631 (50.66)	4139 (5.89)	
Marital status n=186,873	Single	1186 (7.35)	4570 (28.31)	8873 (54.96)	1515 (9.38)	<0.001
	Married	13,846 (10.84)	38,992(30.52)	65,110(50.97)	9792(7.67)	
	Living with a partner	2,202 (7.62)	8,727(30.19)	15,865(54.88)	2116 (7.32)	
	Widowed	281 (11.24)	798 (31.93)	1228 (49.14)	192 (7.68)	
	Divorce	374 (9.48)	1408(35.67)	2002(50.72)	163(4.13)	
	Separated	572 (7.49)	2545 (33.34)	4047(53.02)	469 (6.14)	
Parity n=186,873	Nullipara	2765 (6.79)	11,515(28.28)	22,868(56.16)	3574 (8.78)	<0.001
	Primipara	2862 (7.91)	10,553(29.16)	19,553(54.02)	3226 (8.91)	
	Multipara	7167 (9.82)	22,325(30.59)	37,868 (51.88)	5627 (7.71)	
	Grand multipara	5667 (15.33)	12,647(34.21)	16,836 (45.54)	1820 (4.92)	
Media exposure n=186,873	No	11,728 (16.89)	23,280(33.52)	31,184 (44.91)	3251 (4.68)	<0.001
	Yes	6733 (5.73)	33,760(28.75)	65,941 (56.15)	10,996(9.36)	
Partner Education n=137,749	Illiterate	10,365 (18.72)	18,643(33.67)	23,984 (43.32)	2377 (4.29)	<0.001
	Primary	3246 (7.28)	16,242(36.43)	23,338 (52.35)	1756 (3.94)	
	Secondary	2114 (4.86)	10,526(24.21)	25,564 (58.79)	5277 (12.14)	
	Higher	319 (2.43)	2274(17.43)	8023 (61.23)	2487 (18.98)	

Footnotes: chi-square test was used, ANC: antenatal care.

Perceived distance to healthcare was a key barrier. Among those who reported distance as a major problem, 33.4% (n=23,281) had 1–3 contacts, 46.5% (n=32,426) had 4–7 contacts, and only 5.6% (n=3,896) completed eight or more contacts. In contrast, among those who did not perceive distance as a problem, 28.9% (n=31,620) had 1–3 contacts, 54.9% (n=60,038) had 4–7 contacts, and 9.9% (n=9,763) completed eight contacts (p < 0.001).

A pronounced socio-economic gradient was observed. Among women with no formal education, 33.4% (n=22,360) had 1–3 contacts, 43.0% (n=28,833) had 4–7 contacts, and only 4.6% (n=3,059) completed eight contacts. In contrast, among those with higher education, only 13.1% (n=1,106) had 1–3 contacts, while 61.9% (n=5,227) had 4–7 contacts and 24.0% (n=2,030) completed eight contacts. Among the poorest women, 34.1% (n=15,780) received 1–3 contacts, 43.6% (n=20,217) received 4–7, and 4.0% (n=1,831) achieved eight or more. This contrasts sharply with the richest group, where only 22.4% (n=6,608) had 1–3 contacts, while 60.7% (n=17,909) had 4–7 and 14.5% (n=4,276) completed eight contacts (p < 0.001). Similar trends were observed for residence: among rural women, 33.4% (n=41,942) had 1–3 contacts, 49.2% (n=61,759) had 4–7, and only 5.1% (n=6,392) completed eight. In contrast, urban women had higher coverage at all levels, with 24.6% (n=15,098) receiving 1–3 contacts, 57.6% (n=35,366) 4–7 contacts, and 12.8% (n=7,855) completing eight (Table 1).

### The association between perceived distance and ANC services use

The crude analysis revealed that women who perceived distance to health facilities as a major problem had significantly higher odds of being in lower ANC utilisation categories, compared to those who completed eight or more contacts. Specifically, they had higher odds of receiving 4–7 contacts (cOR = 1.44, 95% CI: 1.33, 1.54, p < 0.001), 1–3 contacts (cOR = 1.97, 95% CI: 1.82, 2.12, p < 0.001), or no ANC at all (cOR = 3.30, 95% CI: 2.98, 3.61, p < 0.001) (Table 2).

**Table 2.** Crude and adjusted multinomial GSEM models assessing the association between perceived distance to healthcare facilities and ANC utilisation (n = 179,162).

​		Base outcomes at least eight ANC contacts					
**Crude model**		4 to 7 contacts		1 to 3 contacts		No	
		cOR (95%CI)	P-value	cOR (95%CI)	p-value	cOR (95%CI)	p-value
Perceived distance to the health care services	A big problem	**1.44(1.33,1.54)**	**<0.001**	**1.97(1.82,2.12)**	**<0.001**	**3.30 (2.98, 3.61)**	**<0.001**
	Not a big problem	1		1		**1**	
**Adjusted model**		aOR(95%CI)	P-value	aOR(95%CI)	p-value	aOR(95%CI)	p-value
Perceived distance to the health care services	A big problem	0.98(0.98,1.06)	0.602	**1.15(1.05,1.25)**	**0.002**	**1.51 (1.35, 1.66)**	**<0.001**
	Not a big problem	1		1		**1**	

Footnote: cOR: crude odds ratio, aOR: adjusted odds ratio, CI: confidence interval, ANC: antenatal care, the model adjusted for residence, maternal age, maternal occupation, maternal education, partner education, parity, household income, media exposure and marital status. Bold text indicates statistical significance at p < 0.01.

After adjusting for socio-demographic and contextual variables, perceived distance remained significantly associated with reduced ANC utilisation. Women who considered distance a big problem had 15% higher odds of receiving only 1–3 contacts (aOR = 1.15, 95% CI: 1.05, 1.25, p = 0.002), and 51% higher odds of receiving no ANC (aOR = 1.51, 95% CI: 1.35, 1.66, p < 0.001), compared to those receiving eight or more contacts. However, the association was no longer significant for women receiving 4 –7 contacts (aOR = 0.98, 95% CI: 0.90, 1.06, p = 0.602) (Table 2).

### The relationship between perceived distance and ANC services utilisation stratified by key socio-demographic characteristics

Overall, the interaction term between perceived distance and key socio-demographic variables was largely not statistically significant across most ANC categories. Specifically, no significant interaction was observed between perceived distance and residence (p=0.91 for 4 to 7 contacts, p=0.63 for 1 to 3 contacts and p=0.08 for no visit), maternal educational status (p=0.048, p=0.15 and p=0.06), wealth index (p=0.09, p=0.15 and p=0.69), and maternal age (p=0.68, p=0.62 and p=0.89, respectively). Whereas the interaction between perceived distance and partner education was statistically significant (p <0.001, p<0.001 and p=0.81, respectively).

Stratified multinomial GSEM models (Table 3), using ≥8 ANC contacts as the reference, showed that perceived distance was consistently associated with higher odds of lower ANC utilisation, particularly among disadvantaged subgroups. Among rural women (n = 104,577), those who reported perceiving distance as a big problem had 17% higher odds of receiving 1–3 contacts (aOR = 1.17, 95% CI: 1.05–1.29, p = 0.003) and 55% higher odds of no ANC (aOR = 1.55, 95% CI: 1.37, 1.74, p < 0.001). Among urban women (n = 46,674), the association was significant only for no ANC (aOR = 1.27, 95% CI: 1.02, 1.53, p = 0.020).

**Table 3.** Adjusted multinomial GSEM models of ANC utilisation stratified by socio-demographic subgroups (n varies).

Rural (n=104,577)							
​		Base outcomes on at least eight ANC contacts					
**Characteristics**		4 to 7 contacts		1 to 3 contacts	​	No	​
		cOR(95%CI)	P-value	cOR(95%CI)	p-value	cOR(95%CI)	p-value
Perceived distance to the health care services	A big problem	0.98(0.89,1.05)	0.688	1.17(1.09,1.25)	**0.003**	**1.55 (1.37, 1.74)**	**<0.001**
**Urban (n=46,674)**							
Perceived distance to the health care services	A big problem	0.98(0.85,1.11)	0.791	1.11(0.94,1.27)	0.170	**1.27 (1.02, 1.53)**	**0.020**
**Lower educational status (n=108,846)**							
Perceived distance to the health care services	A big problem	0.93(0.84,1.02)	0.149	1.10(0.99, 1.21)	**0.056**	**1.48 (1.29, 1.64)**	**<0.001**
**Higher education status (42,405)**							
Perceived distance to the health care services	A big problem	1.03(0.92,1.15)	0.539	1.19(1.04,1.34)	**0.008**	**1.27 (1.05, 1.50)**	**0.007**
**Poor wealth index (70,074)**							
Perceived distance to the health care services	A big problem	0.92(0.82, 1.03)	0.167	1.12(0.98,1.25)	**0.079**	**1.47(1.27,1.68)**	**0.001**
**High Wealth Index (n=50,985)**							
Perceived distance to the health care services	A big problem	1.00(0.88,1.12)	0.982	1.10(0.96,1.25)	0.192	**1.41 (1.15, 1.66)**	**<0.001**
**Lowe partner education (n=97,624)**							
Perceived distance to the health care services	A big problem	**0.87(0.78,0.96)**	**0.001**	1.02(0.91,1.13)	0.647	**1.38 (1.20, 1.56)**	**<0.001**
**Higher partner education (53,627)**							
Perceived distance to the health care services	A big problem	1.07(0.96,1.17)	0.179	**1.27(1.13,1.40)**	**<0.001**	**1.39 (1.18, 1.59)**	**<0.001**
**Young age groups (n=40,565)**							
Perceived distance to the health care services	A big problem	1.00(0.87,1.14)	0.943	**1.25(1.08,1.42)**	**0.002**	**1.55 (1.31, 1.80)**	**<0.001**
**Old age groups (n=39,530)**							
Perceived distance to the health care services	A big problem	0.92(0.81,1.03)	0.193	1.03(0.90,1.16)	0.667	**1.45 (1.23, 1.67)**	**<0.001**

Footnote: Multinomial GSEM models were used with “≥8 ANC contacts” as the reference outcome category. *“Not a big problem”* was the reference group for perceived distance to health facilities. All models were adjusted for relevant covariates. aOR: adjusted odds ratio; CI: confidence interval; ANC: antenatal care. Bold text indicates statistical significance at p < 0.01.

For women with no formal education (n = 108,846), the odds of receiving no ANC were 48% higher (aOR = 1.48, 95% CI: 1.29, 1.64, p < 0.001), with borderline significance for 1–3 contacts (aOR = 1.10, 95% CI: 0.99, 1.21, p = 0.056). Among those with higher education (n = 42,405), perceived distance increased the odds of 1–3 contacts by 19% (aOR = 1.19, 95% CI: 1.04–1.34, p = 0.008) and no ANC by 27% (aOR = 1.27, 95% CI: 1.05–1.50, p = 0.007).

In the lowest wealth group (n = 70,074), perceived distance was associated with 47% higher odds of no ANC (aOR = 1.47, 95% CI: 1.27, 1.68, p = 0.001), while in the highest wealth group (n = 50,985), the increase was 41% (aOR = 1.41, 95% CI: 1.15, 1.66, p < 0.001). Among women with less educated partners (n = 97,624), the odds of no ANC were 38% higher (aOR = 1.38, 95% CI: 1.20–1.56, p < 0.001), and 4–7 contacts were 13% lower (aOR = 0.87, 95% CI: 0.78–0.96, p = 0.001). In the higher partner education group (n = 53,627), distance was associated with 27% higher odds of 1–3 contacts (aOR = 1.27, 95% CI: 1.13–1.40) and 39% higher odds of no ANC (aOR = 1.39, 95% CI: 1.18–1.59, both p < 0.001).

Among younger women (n = 40,565), the odds were 25% higher for 1–3 contacts (aOR = 1.25, 95% CI: 1.08, 1.42, p = 0.002) and 55% higher for no ANC (aOR = 1.55, 95% CI: 1.31, 1.80, p < 0.001). Among older women (n = 39,530), only the no ANC association was significant (45% higher odds, aOR = 1.45, 95% CI: 1.23, 1.67, p < 0.001).

## Discussions

This study is one of the most comprehensive analyses to date, employing GSEM across 26 countries (n = 186,873) to explore the association between perceived distance and ANC services utilisation with a particular focus on the most disadvantaged segments of the population in sub-Saharan Africa. The finding showed that perceiving distance as a major barrier was associated with significantly reduced uptake of recommended ANC services, with 15% higher odds of attending only one to three contacts and 51% higher odds of receiving no contacts compared to completing the recommended eight or more contacts. This effect was more pronounced among less educated women, from low-income households, younger women, and those residing in rural areas. These findings have broader applicability in other low and middle-income countries and refugee settings, where structural and systemic barriers to healthcare access are pervasive and disproportionately affect vulnerable populations.

Our findings are supported by Andersen’s Behavioural Model, which identified geographic accessibility as a key enabling factor influencing individuals’ decisions to seek health services.^27^ Furthermore, the Three-Delay Model highlights a confluence of factors, such as socio-cultural norms, women’s social status, physical remoteness, and limited transport infrastructure, that contribute to delays in deciding to seek prenatal care.^28^ These frameworks help explain the reduced ANC uptake observed in our study, particularly among rural and low-income women who face compounding logistical and social constraints. In our sample, only 5.6% of women who perceived distance as a significant problem completed the recommended eight or more ANC contacts, compared to 9.9% among those who did not perceive distance as a barrier, underscoring the salience of geographic access in shaping maternal healthcare-seeking behaviour. These challenges are especially critical in resource-limited contexts, where high illiteracy rates, inadequate facilities, and economic hardship limit timely and adequate maternal care.^15,31^ One in eight individuals in the region resides more than an hour from the nearest healthcare facility,^32^ while 35.4% of pregnant women report access difficulties and 50.1% cite financial constraints as significant barriers to receiving care. These findings reinforce the need for targeted, multisectoral strategies, such as transport voucher programs, decentralised community-based ANC services, to address both geographic access barriers. Strengthening such locally tailored solutions will be critical to improving uptake of the WHO-recommended ANC model and achieving global goals for maternal health equity and universal health coverage.^12^

In our study, the absence of a significant association among women who attended 4 to 7 ANC contacts suggests that perceived distance may not meaningfully differentiate women at moderate levels of service utilisation from those who achieved at least eight contacts. This finding indicates that once women engage with ANC and reach a moderate number of contacts, geographical distance may play a less decisive role in determining whether they complete the recommended schedule. Other factors, such as health literacy, quality of ANC and health system-related influence, may be more important for at least four contacts.^16,33,34^

The impact of distance on ANC utilisation was especially marked among women from low-income households, those with limited educational attainment, and those whose partners had minimal education. This association may stem from compounding disadvantages linked to poverty, such as unreliable transport, limited disposable income, poor access to health information, and reduced autonomy in decision-making.^34–36^ Women in these circumstances often have constrained agency over healthcare choices,^37,38^ and the indirect costs, transport fares, childcare, and lost income pose significant obstacles to care-seeking.^39,40^ These challenges are further exacerbated in patriarchal societies, where gender norms constrain women’s autonomy and restrict their mobility.^18,19^ In such contexts, male partner influence over health decisions further shapes women’s access to ANC services. Digital exclusion, such as limited access to mobile phones, the internet, or digital health messaging, also contributes to inequities, particularly for women in rural or resource-constrained environments. Moreover, many health systems in under-resourced regions lack the capacity or cultural sensitivity to effectively engage with socio-economically marginalised populations.^41,42^ Failure to access adequate ANC services heightens the risk of delayed detection of pregnancy complications, poor maternal outcomes, and avoidable neonatal morbidity. These overlapping social, financial, technological, and institutional barriers underscore the need to address not only economic and educational inequities but also gendered power dynamics, digital access, and health system responsiveness.

These barriers also affect rural women, and our findings suggest that geographic distance introduces additional challenges. In rural areas, limited transport infrastructure, long travel distances, and poor road conditions often compound barriers such as needing male permission to travel, limited control over household income, or the inability to afford private transport. These constraints are reinforced by social norms that discourage women, particularly younger or unmarried women, from travelling alone.^18^ In our study, rural women who perceived distance as a significant problem had 55% higher odds of receiving no ANC, underscoring the role of physical remoteness as a distinct access barrier.^43^ These challenges are often intensified by the scarcity of health facilities and limited investment in community outreach, making routine ANC less accessible. Expanding midwife-led mobile services and implementing targeted transport support in rural areas may help reduce these inequities.^44^

### Limitations

The strength of this study was the inclusion of a large sample size (n = 186,873), which enabled a robust analysis of the association between perceived distance and ANC utilisation. Additionally, the use of stratified analysis allowed for a focused examination of the most disadvantaged population groups. However, several limitations should be acknowledged. First, both the exposure and outcome variables were based on maternal self-report, which may have introduced recall bias. There is also a potential for social desirability bias in reporting ANC utilisation, especially in contexts where care-seeking is positively reinforced. Second, perceived distance was measured as a binary variable (“big problem” vs. “not a big problem”). This dichotomous and subjective measure may oversimplify the complex geographic and logistical barriers women experience when accessing antenatal care. Because responses are based on individual perception, interpretations of what constitutes a “big problem” may vary across countries and cultural contexts, potentially introducing cross-country heterogeneity and limiting comparability. Third, the cross-sectional design limits the ability to establish causal relationships between perceived distance and ANC utilisation. Temporal and place variation may also be a concern, as the DHS data were collected in different years across the included countries (between 2015 and 2023). Although participants were drawn from different contexts, women in sub-Saharan Africa share common structural barriers to ANC access. However, changes in health policies, service availability, and socio-economic conditions over time may influence comparability across settings. Therefore, findings should be interpreted with caution. The global outbreak of COVID-19 in some survey countries may have affected service utilisation patterns, and this should be considered when interpreting the findings. Furthermore, residual confounding cannot be excluded, given that important factors such as perceived quality of care, local health facility availability, or community norms were not measured. Health system-level characteristics (such as staffing, facility coverage, outreach programs), pre-existing medical problems and pregnancy-related complications were also unavailable in the dataset. Finally, the analysis was restricted to publicly available secondary data, which lacked information on socio-cultural norms and decision-making dynamics that could further elucidate barriers to ANC utilisation. Future research should incorporate these dimensions to more comprehensively examine how structural, cultural, and health system factors interact to influence maternal healthcare access.

## Conclusion

Perceived distance remains a major barrier to ANC utilisation, reflecting deeper structural and social inequities in maternal healthcare access. These findings highlight the urgency of targeted, equity-focused strategies that reach the most affected groups. Strengthening health system responsiveness and addressing mobility, autonomy, and access together will be essential to advancing maternal health outcomes and global health equity.

## Supplemental material

Supplemental material - Effect of perceived distance to health facility on antenatal care service use in Sub-Saharan Africa: Do socio-demographic characteristics modify these associations? Supplemental material for Effect of perceived distance to health facility on antenatal care service use in Sub-Saharan Africa: Do socio-demographic characteristics modify these associations? by Yibeltal Bekele, Ruth L Ngoma, Gedefaw Abeja, Bircan Erbas and Mehak Batra in Women’s Health.

## Data Availability

Data is available upon request on the DHS program website at: https://www.dhsprogram.com/data/available-datasets.cfm.
