# Supplementary material for: Effect of perceived distance to health facility on antenatal care service use in Sub-Saharan Africa: Do socio-demographic characteristics modify these associations?
Source: Womens Health (Lond). 2026 May 13;22:17455057261446938. doi: 10.1177/17455057261446938 (PMC13176555; doi:10.1177/17455057261446938)
Supplement: Supplemental material - Effect of perceived distance to health facility on antenatal care service use in Sub-Saharan Africa: Do socio-demographic characteristics modify these associations? [file sj-pdf-1-whe-10.1177_17455057261446938.pdf]

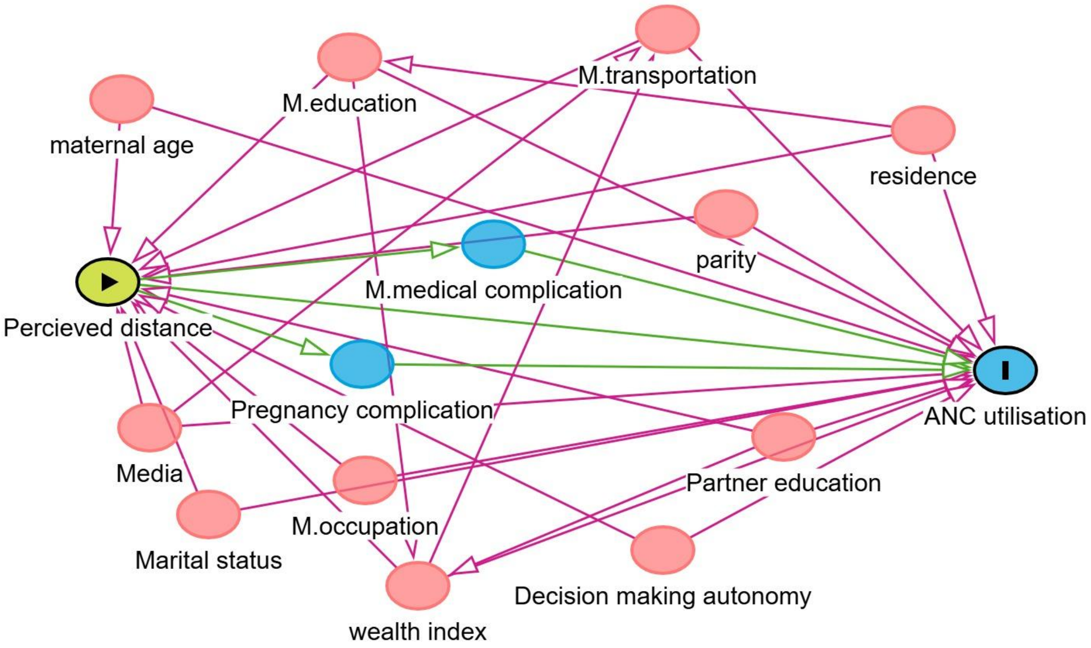

Figure S1. Direct acyclic graph used to understand the causal pathways: ANC: antenatal care, M.education, m.occupation: maternal occupation, M.education: maternal education, M.medical complications: maternal medical complication.
